# Evaluating a cardiovascular disease risk management care continuum within a learning healthcare system: a prospective cohort study

**DOI:** 10.3399/bjgpopen20X101109

**Published:** 2020-11-18

**Authors:** T Katrien J Groenhof, A Titia Lely, Saskia Haitjema, Hendrik M Nathoe, Marlous F Kortekaas, Folkert W Asselbergs, Michiel L Bots, Monika Hollander

**Affiliations:** 1 Julius Center for Health Sciences and Primary Care, University Medical Center Utrecht, Utrecht, The Netherlands; 2 Wilhelmina Children’s Hospital Birth Center, University Medical Center Utrecht, Utrecht, The Netherlands; 3 Laboratory of Clinical Chemistry and Haematology, University Medical Center Utrecht, Utrecht, The Netherlands; 4 Department of Cardiology, Division Heart & Lungs, University Medical Center Utrecht, Utrecht, The Netherlands; 5 Institute of Cardiovascular Science, Faculty of Population Health Sciences, University College London, London, UK; 6 Health Data Research UK, Institute of Health Informatics, University College London, London, UK

**Keywords:** learning healthcare system, continuity of patient care, cardiovascular risk management, cardiovascular diseases

## Abstract

**Background:**

Many patients now present with multimorbidity and chronicity of disease. This means that multidisciplinary management in a care continuum, integrating primary care and hospital care services, is needed to ensure high quality care.

**Aim:**

To evaluate cardiovascular risk management (CVRM) via linkage of health data sources, as an example of a multidisciplinary continuum within a learning healthcare system (LHS).

**Design & setting:**

In this prospective cohort study, data were linked from the Utrecht Cardiovascular Cohort (UCC) to the Julius General Practitioners' Network (JGPN) database. UCC offers structured CVRM at referral to the University Medical Centre (UMC) Utrecht. JGPN consists of electronic health record (EHR) data from referring GPs.

**Method:**

The cardiovascular risk factors were extracted for each patient 13 months before referral (JGPN), at UCC inclusion, and during 12 months follow-up (JGPN). The following areas were assessed: registration of risk factors; detection of risk factor(s) requiring treatment at UCC; communication of risk factors and actionable suggestions from the specialist to the GP; and change of management during follow-up.

**Results:**

In 52% of patients, ≥1 risk factors were registered (that is, extractable from structured fields within routine care health records) before UCC. In 12%–72% of patients, risk factor(s) existed that required (change or start of) treatment at UCC inclusion. Specialist communication included the complete risk profile in 67% of letters, but lacked actionable suggestions in 86%. In 29% of patients, at least one risk factor was registered after UCC. Change in management in GP records was seen in 21%–58% of them.

**Conclusion:**

Evaluation of a multidisciplinary LHS is possible via linkage of health data sources. Efforts have to be made to improve registration in primary care, as well as communication on findings and actionable suggestions for follow-up to bridge the gap in the CVRM continuum.

## How this fits in

Multimorbidity, high complexity, and chronicity of disease require multidisciplinary management in a care continuum. Preferably, care evaluations should incorporate the entire continuum, including primary and hospital care. The CVRM continuum from GP to hospital and vice versa was evaluated via linkage of health data sources, as an example of a complex multidisciplinary care continuum. Efforts have to be made to improve registration of cardiovascular risk factors in primary care, as well as communication on findings and actionable suggestions for follow-up from specialists to GPs to bridge the gap in the CVRM continuum.

## Introduction

Modern medical practice takes place in the context of a growing and ageing population, which means it is characterised by multimorbidity, high-complexity diseases, and a high proportion of chronic diseases.^1,2^ In order to provide high quality care, multidisciplinary collaboration and communication between all different caregivers is needed to form a care continuum.^3^ Evidence for clinical practice is derived from medical research. Classically, medical research is conducted outside routine practice in randomised controlled trials and dedicated cohorts with strict inclusion and exclusion criteria.^4^ Because the real-world patient does not continuously fit these criteria, generated evidence does not always translate to daily practice.^5^ Within a learning healthcare system (LHS), data from daily practice is used as input for analysis, interpretation, and feedback.^6^ Compared with conventional medical research, LHS-based research is potentially more efficient in both time and costs, and is not hampered by selection and decreased generalisability owing to strict inclusion and exclusion criteria. Preferably, the LHS incorporates the entire care continuum.^4,7^

Cardiovascular diseases (CVD) are a good example of high complexity, multimorbid, and chronic diseases.^8^ Prevention of CVD is basically the same for all patients: lifelong cardiovascular risk factor management (CVRM). In the Netherlands, GPs usually have a longstanding relationship with their patients. Therefore, chronic disease management, such as CVRM, is placed in their portfolio. CVRM guidelines provide various recommendations for diagnosis, treatment, and referral. If necessary, patients are referred to secondary or tertiary care for further evaluation of their cardiovascular condition. The responsibility of the CVRM can be transferred to a hospital specialist, and back to the GP after cessation of hospital care.^9^ Together, all caregivers contribute to a multidisciplinary CVRM continuum.

The aim of this study was to evaluate the CVRM continuum via linkage of health data sources, as an example of a multidisciplinary LHS.

## Method

### Study design 

A prospective cohort study was conducted with data from the ongoing UCC and JGPN.^10,11^

### Data source

The Centre of Circulatory Health of the University Medical Centre (UMC) Utrecht initiated the UCC in 2016. In short, UCC is an ongoing prospective cohort study, collecting routine clinical data from patients referred for a CVD (risk factor) to the UMC Utrecht.^10^ Informed consent for linkage with external parties was obtained in the UCC. The UCC has been approved by the local Institutional Review Board.

The JGPN database consists of routine care data from over 10 years of a dynamic cohort of around 370 000 individuals registered with the participating GPs from the city of Utrecht and its vicinity.^11^ Informed consent was waived for JGPN participation based on the 'law on the medical consultation' (the Dutch Medical Treatment Contracts Act [WGBO]) exception rules for research with medical care data where: (i) patients are anonymous; (ii) there is no breach of personal integrity; (iii) research cannot be performed without this data; (iv) research serves a common benefit; and (v) patients are informed on the usage, are provided an opportunity to opt-out, and do not explicitly object.^12^ All JGPN practices were obliged to inform their patients on the JGPN database and the opt-out procedure.^11^

### Participants 

All UCC patients that provided informed consent for linkage with third party registries that were also part of the JGPN database were eligible for this analysis. Information was requested on the cardiovascular risk profile — smoking, alcohol use, body mass index (BMI), blood pressure (BP), lipids, glucose, renal function, physical activity, cardiovascular history, and medication — for each patient between 13 months before referral to the UMC Utrecht (JGPN), at referral (UCC), and 12 months after referral (JGPN).^9^

To combine UCC and JGPN data sources, the UCC patients were linked to their JGPN records at an individual patient level using a trusted third party (TTP). The TTP received UCC pseudo-IDs plus identifying information and matched them with JGPN pseudo-IDs. TTP supplied the JGPN data manager with JGPN pseudo-IDs, who added requested JGPN variables to the dataset. This was sent back to TTP, who then removed JGPN pseudo-IDs and added UCC pseudo-IDs. This dataset was provided to the UCC data manager, who then added requested UCC variables to the set and provided the complete dataset to the researchers.

### Measurement characteristics 

JGPN data were extracted from structured fields within the GPs’ EHRs. Prescribed cardiovascular medication was extracted from the electronic prescription system via predefined Anatomical Therapeutic Chemical codes (Supplementary Table S1). UCC data were collected via a questionnaire, biometric measurements, and blood drawn in routine care at the UMC Utrecht, all registered in predefined fields within the EHR.^10^ All variables and their source in JGPN and UCC are listed in Supplementary Table 2.

### Outcome measurements

#### Risk factor registration

First, the registration of the risk factors were assessed: smoking, alcohol use, BMI, systolic blood pressure (SBP), low-density lipoprotein cholesterol (LDL-c), glucose, renal function, and physical activity in JGPN before and after UCC inclusion. The variable *'at least one risk factor registered in JGPN'* was defined as *'yes'* if at least one of these factors was registered in JGPN. Differences in individual risk factor levels were compared between patients with any risk factor registered to patients with *'none'* of the risk factors registered in JGPN before referral to UCC.

#### Risk factor target attainment

Second, based on the UCC risk profile, patients were stratified according to the European Society of Cardiology (ESC) risk categories.^8^ Then the patient’s target status — either on or off target — was determined dependent on their risk category. The BP target was below 140/90 mmHg in every risk category. The LDL-c target was <1.8 mmol/l for the very high-risk category and <2.5 mmol/l for the high-risk category. The HbA1c target was <53 mmol/l for patients with type 2 diabetes.

#### Added value of UCC to detect risk factors with indication for treatment

Third, the added value of UCC to detect hypertension, dyslipidaemia, and diabetes was assessed. The added value was defined as the proportion of patients with a de novo condition detected in the UCC plus the patients with known conditions but off-target measurements. Patients without reported hypertension, dyslipidaemia, or diabetes but with an off-target measurement were defined as *'newly diagnosed'*. For diabetes, the threshold for newly diagnosed diabetes was an HbA1c>48 mmol/mol.

#### Communication between the specialist and the GP

Fourth, in the specialist letter after the UCC consult, the level of completeness of the cardiovascular risk profile was evaluated, and if the specialist specifically advised follow-up action(s) for CVRM.

#### Follow-up

Last, follow-up and change of CVRM after the UCC consult of BP, LDL-c, and HbA1c were assessed. Change of CVRM was defined as a change in the absolute level of the risk factor and/or a change in medication prescription when comparing medication use reported in JGPN before and UCC to the follow-up measurement. The authors defined positive change (risk factor level decreased and/or medication was commenced or changed), stable off target (no change in management and still off target), and stable on target (no change in management, still on target).

### Statistical analyses

Statistical analyses were conducted in R studio (version 3.4.1). Student *t*-tests were used to compare normally distributed continuous variables and Pearson's χ^2^ or Fisher’s exact test where appropriate for proportions.

## Results

### Participants

**Figure 1.**
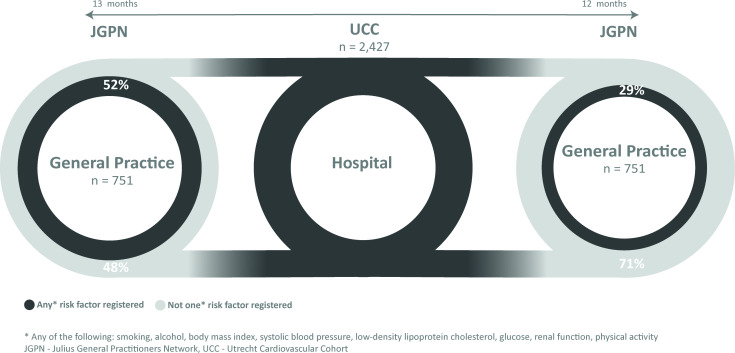
Flowchart of patient selection and risk factor assessment overview

Out of 2427 UCC patients (included from January 2016–14 May 2019), 751 (31%) could be identified in the JGPN database (Figure 1), of which 231 (31%) were at high risk and 520 (69%) at very high risk for CVD, according to the ESC classification.^8^ All patients had an indication for annual CVRM check-up.^8^ In 112 patients (15%) the GP was listed as the lead caregiver for CVRM, in 25 (3%) the (cardiovascular) specialist, and in 614 (82%) no lead caregiver was registered in the JGPN database.

### The CVRM continuum

#### Risk factor registration

Before UCC, ≥1 risk factors were registered for 392 patients in JGPN (52%) (Figure 1). Patients’ height (6%) was registered the least (10%), SBP the most (43%) (Table 1). Patients with registered risk factors in JGPN before UCC inclusion showed a more unfavourable risk profile (Table 2): mean age, BMI, and SBP were higher and estimated glomerular filtration rate (eGFR) was lower compared with patients without registered risk factors. In both groups similar proportions were found of women and patients with coronary heart disease, chronic heart failure, stroke, and peripheral artery disease.

**Table 1.** Percentage of registration of factors in JGPN

**Risk factors**	**Before UCC (%**), total *n* =751	**After UCC (%**), total *n* = 751
Smoking	27	17
Alcohol use	21	13
Height	6	4
Weight	27	19
BMI	25	17
Family history of CVD <60 years	10	6
Physical activity	20	13
Systolic blood pressure	43	29
Diastolic blood pressure	43	29
HDL-cholesterol	37	21
LDL-cholesterol	36	21
Triglycerides	37	21
Total cholesterol	37	21
Glucose (fasting or non-fasting)	43	27
eGFR	46	27
Number of risk factors available^a^		
0	48	71
1–3	28	22
4–6	24	15

^a^From: smoking, alcohol, BMI, systolic blood pressure, low-density lipoprotein, glucose, renal function, physical activity.

JGPN Julius General Practitioners' Network UCC Utrecht Cardiovascular Cohort BMI body mass index CVD cardiovascular disease HDL high-density lipoprotein LDL low-density lipoprotein eGFR estimated glomerular filtration rate

**Table 2.** Risk factor profile measured at UCC inclusion

	**None of RFX registered in JGPN before referral** ***n* = 359** (**48%**)	**At least one of RFX registered in JGPN before referral** ***n* = 392** (**58%**)
**Mean age, years** **(SD)**	52 (18)	65 (16)
**Women*, n*** **(%)**	190 (53)	189 (48)
**Anthropometry**		
Mean BMI kg/m^2^ (SD)	25 (5)	28 (6)
Mean height, cm (SD)	174 (14)	171 (11)
Mean weight, kg (SD)	78 (18)	80 (18)
**Lifestyle**		
Currently smokes (missing 12%)	43 (14)	38 (11)
Current alcohol use (missing 12%)	192 (63)	188 (53)
Physical activity, minutes per week (missing 25%)	1881 (1285)	1666 (1233)
**Laboratory measurements**		
Mean systolic blood pressure, mmHg (SD)	133 (20)	146 (24)
Mean diastolic blood pressure, mmHg (SD)	79 (12)	81 (13)
HDL-cholesterol	1.4 (0.4)	1.4 (0.5)
LDL-cholesterol	3.2 (1.3)	3.1 (1.3)
Triglycerides	1.6 (1.2)	2.0 (2.4)
Total cholesterol	5.3 (1.5)	5.3 (1.6)
Mean eGFR (1.73/ml/min, [SD])	91 (25)	79 (21)
Mean HbA1C, mmol/mol (SD)	38 (10)	53 (12)
**History of CVD**		
Coronary heart disease	28 (7.8)	83 (21)
Chronic heart failure	28 (7.8)	30 (7.7)
Stroke	34 (9.5)	65 (16.6)
Peripheral artery disease	19 (5.3)	46 (12)

RFX risk factor JGPN Julius General Practitioners' Network UCC Utrecht Cardiovascular Cohort BMI body mass index CVD cardiovascular disease HDL high-density lipoprotein LDL low-density lipoprotein eGFR estimated glomerular filtration rate HbA1c glycated haemoglobin

#### Risk factor target attainment, added value of UCC, and follow-up: blood pressure

**Figure 2.**
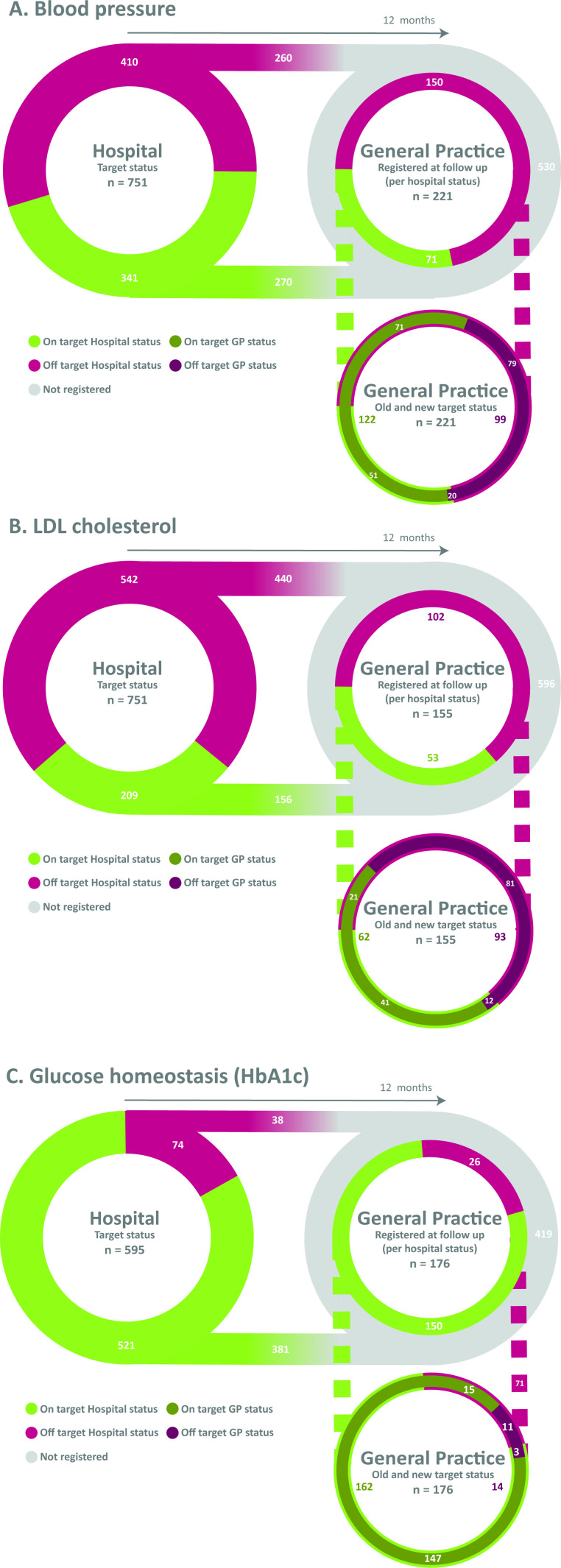
The multidisciplinary care continuum for blood pressure, LDL-cholesterol, and glucose management

In UCC, 410 (55%) patients required change or start of BP lowering treatment (off-target measurement) (Figure 2A). Of the 751 patients, ≥1 risk factors were registered in 221 (29%) patients at follow-up in JGPN after UCC. More patients who were off target were followed-up (150 out of 410, 37%) compared with those who were on target (71 out of 341, 21%). Of patients who were off target, 71 (47%) improved at follow-up.

#### Risk factor target attainment, added value of UCC and follow-up: LDL-cholesterol

In UCC, 542 (72%) patients required change or commencement of LDL-c lowering treatment (off-target measurement) (Figure 2B). Of the 751 patients, ≥1 risk factors were registered in 155 (21%) patients at follow-up in JGPN after UCC. Fewer patients who were off target were followed-up (102 out of 542, 19%) compared with those who were on target (53 out of 209, 25%). Of patients who were off target, 21 (21%) improved.

#### Risk factor target attainment, added value of UCC and follow-up: blood glucose

In UCC, 74 (of 595, 12%) patients required change or commencement of blood glucose lowering treatment (off-target measurement or new diagnosis of diabetes mellitus) (Figure 2C). Of the 595 patients, 176 (30%) were followed-up. More patients who were off target were followed-up in JGPN after UCC (26 out of 74, 35%) compared with those who were on target (150 out of 521, 29%). Of patients who were off target, 15 (58%) improved.

#### Communication between the specialist and the GP

Specialist letters to the GP were assessed in a subset of 311 patients. The most frequent reasons for referral to the specialist were analysis of coronary heart disease (*n* = 74, 20%) and analysis of cognitive impairment (*n* = 60, 19%). All patients were referred to the UMC Utrecht by their GP. The specialist of referral reported back to the GP on 95% of the consults. In 8% of these letters, none of the cardiovascular risk factors were reported, in 29% one or more were missing (mostly lipids), and in 63% a complete risk profile was reported. The CVRM profile was more often reported in patients in whom risk factor management required a change, that is, changing of treatment or starting treatment. In 43 letters (14%), the specialist specifically suggested follow-up action(s) for the cardiovascular risk factors by the GP (such as: *'* *please follow-up* *blood pressure* *'*), implying a leadership role for the GP regarding CVRM.

## Discussion

### Summary

In this study the patient trajectory in the CVRM continuum was evaluated as an example of a multidisciplinary LHS. Structured assessment of the risk profile in tertiary care has added value: many patients required (start or change of) treatment of a risk factor. Yet, only a few specialists specifically highlight the CVRM in their letter to the GP and follow-up in general practice might, therefore, be lacking. Based on the combined hospital and GP data, it is unclear in most patients who is lead caregiver regarding CVRM.

### Strengths and limitations

The presented project was a proof-of-concept of how a multidisciplinary LHS can be evaluated via linkage of data sources. Because the population is a tertiary centre population, 31% of patients were from the Utrecht area and could be linked. If more data sources would be made more easily linkable, such as other hospital data and/or pharmacy data, this would improve some of the follow-up gaps, and increase power of the analyses. It could very well be that (part of) this high-risk population were under follow-up in another hospital. Furthermore, the study was restricted to patients who provided informed consent for linkage of UCC to external parties.^13^ For an LHS, current ethical and legislative frameworks do not suffice because they are based on the classical separation of science and care. Initiatives that focus on the design of a new framework, which empowers LHS developments but safeguards integrity of patients, are arising.^14^

Specifically to CVRM, the timeframe for follow-up was discussed extensively. Since new medication should be evaluated within 3 months,^9^ the study should have caught this follow-up visit in the extraction. Also, the minus 13 to plus 12 months range allowed information on yearly CVRM to be retrieved.

To construct target attainment prevalence for hypertension, dyslipidaemia, and diabetes above target BP, LDL-c, and HbA1c were defined as absolute measurements in combination with the ESC-risk classification. However, the guidelines also allow for a relative target attainment; for example, an LDL-c reduction of 50% compared with the first measurement.^15^ Because the authors did not have information on the first measurement on which the diagnosis was defined, they could not construct these relative target attainment measures. This might have resulted in false off-target classification.

### Comparison with existing literature

In the analysis, the study found room for improvement in registration and communication of CVRM. Organised identification of eligible patients is essential for the establishment of a care continuum. Studies on cardiac rehabilitation confirm this hypothesis: uptake of rehabilitation programmes is highly dependent on the identification of eligible patients after manifestation of the event.^16,17^ Structured registration of the risk factors in all EHRs (GP or hospital), enabling automated extraction of relevant information of a specific patient population, is essential for an LHS. In the UCC hospital identification of eligible patients was organised and uniformity of registration was safeguarded by providing an organisational structure to all departments providing care for patients with CVDs.^10^ Yet, registration of data in JGPN is not uniformly organised and based on structured field-only extractions. It is known that much of clinically relevant data are still registered in unstructured clinical notes.^18^ Structured collection and registration similar to UCC and also more advanced methods, such as data mining of free text, could be incorporated to improve data completeness and quality.^18^ Lastly, communication between specialists and GPs mostly occurs through letters, yet recommendations on the next steps in treatment or follow-up and even vital information are frequently absent in these letters.^19,20^ Structured and timely communication between caregivers is essential for continuity of care and is associated with fewer adverse outcomes.^21^ Communication should be standardised and at least contain factor levels including interpretation, suggestions for follow-up and (transfer of) CVRM leadership. Potential solutions are a template letter or automatically generated letters.^21^

### Implications for research and practice

In conclusion, evaluation of a multidisciplinary transmural LHS is possible via linkage of health data sources. The results indicate that structured assessment of risk factors has added value for detecting risk factor(s) requiring treatment. Organised identification of eligible patients, structured registration in primary care and secondary care in structured fields, as well as communication on findings and actionable suggestions for follow-up need to be improved to solve the gap in the CVRM continuum. The authors suggest that the GP can coordinate this continuum through strong leadership.
